# Development and validation of an environmental heat strain risk assessment (EHSRA) index using structural equation modeling based on empirical relations

**DOI:** 10.1186/s12199-020-00894-1

**Published:** 2020-10-28

**Authors:** Saeid Yazdanirad, Farideh Golbabaei, Abbas Rahimi Foroushani, Mohammad Reza Monazzam, Habibollah Dehghan

**Affiliations:** 1 Department of Occupational Health Engineering, School of Public Health, Tehran University of Medical Sciences, Tehran, Iran; 2 Department of Epidemiology and Biostatistics, School of Public Health, Tehran University of Medical Sciences, Tehran, Iran; 3 Department of Occupational Health Engineering, School of Public Health, Isfahan University of Medical Sciences, Isfahan, Iran

**Keywords:** Heat stress, Risk assessment, Structural equation modeling, Empirical index

## Abstract

**Background:**

Need to a simple, available, accurate, comprehensive, and valid indicator is felt to assess thermal effects. Therefore, the present study was aimed to develop and validate the environmental heat strain risk assessment (EHSRA) index using structural equation modeling (SEM) based on empirical relations.

**Methods:**

This cross-sectional study was performed on 201 male workers in environments with various climatic conditions. The heart rate and tympanic temperature of the individuals were monitored at times of 30, 60, and 90 min after beginning the work. At these times, values of dry temperature, wet temperature, globe temperature, and air velocity were also measured and metabolism rate and clothing thermal insulation value were estimated. At the end, a theoretical model was depicted in AMOS software and obtained coefficients were applied to develop a novel index. The scores of this indicator were categorized into four risk levels via ROC curves and validate using linear regression analysis.

**Results:**

Indirect effect coefficients of the globe temperature, dry temperature, wet temperature, air velocity, metabolism, and clothing thermal insulation variables on the tympanic temperature were computed by 0.77, 0.75, 0.69, 0.24, 0.49, and 0.39, respectively. These coefficients were applied to develop the index. Optimal cut-off points of boundaries between risk levels included 12.02, 15.88, and 17.56. The results showed that the EHSRA index justified 75% of the variations of the tympanic temperature (*R*^2^ = 0.75).

**Conclusions:**

The novel index possesses appropriate validity. It was suggested that this indicator is applied and validated in various environments in the next studies.

## Background

People occupied in industrial environments are exposed to many hazards to threaten their health. Heat is one of the most prominent physical harmful agents in workplaces. Nearly 40% of the world’s people live in the warm and hot climatic regions of the earth, where normal daytime temperatures are higher than 30 °C at most days of a year [1]. In these zones, numerous workers are performing routine tasks. Global warming will increase adverse thermal effects on bodily health [2]. The results of several studies show that air temperature may raise from 1.1 to 6.4 °C at the end of the twenty-first century compared to the nineteenth century [3]. Additionally, processes in some industries such as cement, steel, casting, and food produce and diffuse heat because of energy conversions and weak insulation [4]. The elevated temperature associated with humid conditions and intense physical activity also increases the risk [5]. The excessive exposure of workers can cause health problems such as fatigue, headache, muscle cramps, weakness, dizziness, nausea, vomiting, tachycardia, hypotension, syncope, progressive loss of mental alertness, stroke, and even death [6]. Although consequences of work under hot and humid conditions threat bodily health in developed countries, this problem is much more serious in developing and low- to middle-income countries such as Iran because of the existence of aged industries, weak control measures, densely populated environments, high physical workloads, and weak safety regulations [7].

To prevent heat effects, the fundamental effective agents in producing thermal strain must be assessed, so that through controlling them heat-related illnesses can be reduced. In total, the body heat balance is determined by six substantial factors, including four climatic )dry temperature, radiant temperature, humidity rate, and air velocity( and two non-climatic )clothing and physical workload( variables [8]. Imbalance of these factors impresses on physiological parameters, so-called thermal strain, and results in the occupational illnesses [9]. In fact, the body dissipates heat through dry and evaporative losses [10]. An environment with high air and radiant temperatures disrupt the dry mechanism and an ambiance with excessive humidity impairs the evaporative mechanism. Therefore, the heat produced by physical activity cannot easily be transferred from the body to the external environment [5]. The air movement also moderates this exchange. The effect of air velocity depends on dry temperature so that if the temperature is lower than skin temperature, increased air velocity reduces the strain, otherwise enhances it [11]. Moreover, clothing characterization such as thermal insulation and evaporative resistance influence this balance and can limit the transition [12]. An index must consider all stated six factors for comprehensively and appropriately assessing the occupational heat stress [13]. Many indicators have been designed to evaluate it. Freitas and Grigorieva conducted a study to identify and investigate numerous thermal climate indices and ultimately found 165 indicators, of which a few of them such as predicted heat strain (PHS) involve all six variables [14]. Other properties of a desirable index include simplicity, availability, accuracy, validity, reliability, and repeatability. Havenith and Filad investigated 35 heat stress indicators and models. They concluded that simple indices are most popular in the field and complex models appear to be limited in use [15]. Rational indicators such as PHS involve complex equations for calculating thermal strain while empirical indices possess more simplicity. Additionally, an index must have good validity and reliability for predicting the thermal strain. Wet bulb global temperature (WBGT), as an empirical indicator, is among the most widely used in many countries and there is an international standard of ISO 7243 based on it [16]. However, this index is being outdated after 60 years of use and has been criticized by some authors [17]. As well as many indices have not appropriately categorized and interpreted the risk of thermal strain. Need to a simple, available, accurate, comprehensive, and valid indicator is felt. Therefore, the present study was aimed to develop and validate the environmental heat strain risk assessment (EHSRA) index using structural equation modeling (SEM) based on empirical relations.

## Methods

### Participants

This cross-sectional study was performed on 201 male workers, including 111 subjects from a steel industry located in Isfahan province of Iran as a hot and dry environment and 90 persons from a petrochemical industry placed in Bandar abbas province of Iran as a hot and humidity ambiance. The main difference between the two industries was the climatic conditions so that the petrochemical industry had higher relative humidity. After visiting various departments in each of the above industries, desired tasks and workstations were carefully selected based on the environmental parameters and physical workload. Subsequently, the 400 individuals employed in these parts were invited to the study and evaluated. Based on the criteria, 199 persons were excluded and 201 subjects remained in the study. Inclusion criteria consisted of work experience higher than one year in a warm environment, absence of mental, infectious, pulmonary, cardiovascular, hypertension, renal, hyperthyroidism, digestive, and diabetes diseases, absence of musculoskeletal disorders, non-consumption of medications affecting heart rate and blood pressure such as beta-blockers, phenothiazines, diuretics, anticholinergics, antispasmodics, psychotropics, antihistamines, antihypertensives, amphetamine, and decongestants, and non-consumption of coffee, caffeine, and alcohol from 12 h before the study. Additionally, their tympanic membrane and auditory canal were in a pleasant situation and lack of excessive wax. Exclusion criteria for participants in this research included unwillingness to impressively continue, lack of cooperation in accurate measurement of physiological parameters, heart rate higher than the value calculated in Eq. 1 (HRmax), and tympanic temperature above 39 °C.

1 $$ \mathrm{HRmax}=\left[208-\left(0.7\times \mathrm{age}\ \left(\mathrm{year}\right)\right)\right] $$

### Data collection

The protocol of this study was reviewed and approved by the Medical Ethics Committee of Tehran University of Medical Sciences (IR.TUMS.SPH.REC.1397.321). Each of the participants was invited to refer to a cool and quiet room at a certain time. In the referral day, the person filled out the consent form developed by the medical ethics committee, and his demographical data, including age, workstation, physical activity, and work experience was collected. Height and weight were carefully measured using a tape meter and a digital scale. After that, the individual was invited to rest and relax on a bed in the room for 30 min. His heart rate and tympanic temperature were properly measured at times of 20, 25, and 30 min based on the standard of ISO 9886. At the next step, the person was asked to return to his workplace and start the routine tasks without resting during the test. The physiological parameters of the individual were accurately monitored at times of 30, 60, and 90 min after beginning the work based on the standard of ISO 9886. At these times, environmental climatic parameters, including dry temperature (*T*_a_), wet temperature (*T*_w_), globe temperature (*T*_g_), and air velocity (*V*_a_) were also measured based on standards of ISO 7243 and ISO 7726. As well as, the mean value of metabolism rate (*M*_t_) was estimated by the standard of ISO 8996 and corrected by the standard of BS 7243. To determine clothing thermal insulation (*I*_c_), the standard of ISO 9920 was applied. It should be noted that each of the thermal and physiological variables was recorded at three times. The mean values of thermal variables and the highest values of physiological variables were considered as representative values.

### Instruments

The tympanic temperature, as the gold standard, was measured using a tympanic thermometer of Braun thermoscan IRT 6530 with an accuracy of 0.1 degrees of centigrade. This device has been registered as a patent with pre-warmed tips and an exact positioning system, which increases the validity and accuracy of measurements. Various studies investigated the reliability of this thermometer for measuring the tympanic temperature. Navarro et al. concluded Braun thermoscan IRT 6530 reliably estimates the body temperature during exercise in the heat [18]. To measure the heart rate, pulse monitor with chest strap of Beurer PM70 model with an accuracy of one beat per minute was also applied. Environmental climatic parameters, including dry temperature, wet temperature, and globe temperature were monitored using WBGT meter of TES 1369B with an accuracy of 0.1° of centigrade. The air velocity was separately screened using TES 1340 with an accuracy of 0.01 m/s. Height and weight were also measured using a tape meter with an accuracy of 0.01 m and a digital scale of Hamilton with an accuracy of 0.1 kg, respectively.

### Index development

After designing the model in AMOS software, the indirect effect values of six main parameters in changing tympanic temperature were used as variable coefficients in the novel index. Before writing the final formula, each of the variables was normalized between 0 and 1 based on values measured in the present study. Equation 2 shows the relation used to compute the novel index.

2 $$ \mathrm{EHSRA}=\left[\left({C}_1\times {T}_{an}\right)+\left({C}_2\times {T}_{wn}\right)+\left({C}_3\times {T}_{gn}\right)+\left({C}_4\times {V}_{an}\right)+\left({C}_5\times {M}_{tn}\right)+\left({C}_6\times {I}_{cn}\right)\right] $$

Where *C*_1_ to *C*_6_ shows effect coefficients of fundamental variables, *T*_*an*_ is normalized dry temperature, *T*_*wn*_ is normalized wet temperature, *T*_*gn*_ is normalized globe temperature, *V*_*an*_ is normalized air velocity, *M*_*tn*_ is normalized total metabolism, and *I*_*cn*_ is normalized clothing thermal insulation.

### Statistical analyses

Ultimately, data were entered into the statistical package for the social sciences (SPSS) version 18. Descriptive analyses were performed. After that, the normality of variables was investigated using skew and kurtosis curves in AMOS. Based on the results, all of them showed normal distributions. Hence, correlations were calculated using the Pearson test. Based on the relationships among variables, an empirical model was depicted in AMOS software. Its fitness was evaluated using three groups of absolute, comparative, and normed fit indices. In the model, factor loading values of critical variables on heat strain (latent variable) and tympanic temperature (gold standard variable) were calculated. Subsequently, based on these results, the novel index was developed. In final, the total score of the index was categorized into four risk levels from low to very high using Receiver operator curves (ROC) analysis. In ROC analysis, tympanic temperatures of 37.5, 38.0, and 38.5 ^°^C were considered as boundaries between risk levels [19]. To discover optimal cut-off points of the index, the nearest point to the ideal state in each of the ROC curves was used. To investigate the validity of the index, a linear regression analysis was performed between the EHSRA index and tympanic temperature.

## Results

Table 1 represents the local climatic information. Moreover, Table 2 shows the statistical distribution of demographics parameters, fundamental factors, and physiological parameters. Skew and kurtosis curves revealed that the studied variables had normal distributions. Based on the results, each of the variables encompassed the extensive range of values that the model requires. As well as, the fundamental factors could produce the variations in tympanic temperature from 36.7 to 39.1 °C. Table 3 also reports the correlation matrix of the studied variables. There were significant relationships between tympanic temperature and all fundamental factors (*P* < 0.01). The highest correlation coefficients with tympanic temperature were related to variables of the globe, dry, and wet temperatures with values of 0.766, 0.751, and 0.685, respectively.

**Table 1:** The local climatic information

Parameter	Steel industry			Petrochemical industry		
	Range	Mean	Standard deviation	Range	Mean	Standard deviation
Dry temperature (degree of centigrade)	21.97–43.60	33.58	5.21	24.10–48.20	36.26	6.61
Wet temperature (degree of centigrade)	12.10–24.17	17.63	2.04	13.97–37.57	27.43	6.26
Globe temperature (degree of centigrade)	23.40–62.43	39.01	9.63	24.10–57.23	40.97	9.80
Relative humidity (%)	9.01–39.31	19.60	9.92	14.82–79.11	52.09	17.71

**Table 2:** Statistical distribution of demographics parameters, fundamental factors, and physiological parameters

Variable		Range	Mean	Standard deviation
Demographics parameters	Age (years)	22–55	36.62	8.24
	Weight (kg)	55.70–123.00	80.52	14.91
	Height (m)	1.61–1.90	1.76	0.06
	Body mass index (kilogram per square meter)	19.23–34.94	26.06	4.07
	Physical activity (hours per week)	0–12	2.14	2.47
	Work experience (year)	1–40	12.72	7.85
Fundamental factors	Dry temperature (degree of centigrade)	21.97–48.20	24.78	6.01
	Wet temperature (degree of centigrade)	12.10–37.57	22.01	6.60
	Globe temperature (degree of centigrade)	23.40–62.43	39.89	9.73
	Air velocity (meter per second)	0–4.20	0.58	0.39
	Total metabolism (watts)	130–490	248.47	103.70
	Clothing thermal resistance (clo)	0.50–1.35	0.83	0.14
Physiological parameters	Resting tympanic temperature (degree of centigrade)	36.7–37.4	37.01	0.15
	Working tympanic temperature (degree of centigrade)	36.7–39.1	37.7	0.56
	Resting heart rate (beat/min)	64.00–94.00	76.14	5.99
	Working heart rate (beat/min)	70.00–189.00	121.00	28.45

**Table 3:** Correlation matrix of the studied variables

Variables	1	2	3	4	5	6	7
Dry temperature	–						
Wet temperature	0.626^**^	–					
Globe temperature	0.907^**^	0.511^**^	–				
Air velocity	0.296^**^	0.152^*^	0.347^**^	–			
Total metabolism	0.318^**^	0.131	0.332^**^	0.082	–		
Clothing thermal insulation	0.454^**^	0.054	0.530^**^	0.135	0.321^**^	–	
Tympanic temperature	0.751^**^	0.685^**^	0.766^**^	0.258^**^	0.509^**^	0.388^**^	–

(1) Dry temperature, (2) wet temperature, (3) globe temperature, (4) air velocity, (5) total metabolism, (6) clothing thermal insulation, (7) tympanic temperature

^**^P < 0.01

^*^P < 0.05

**Fig. 1:**
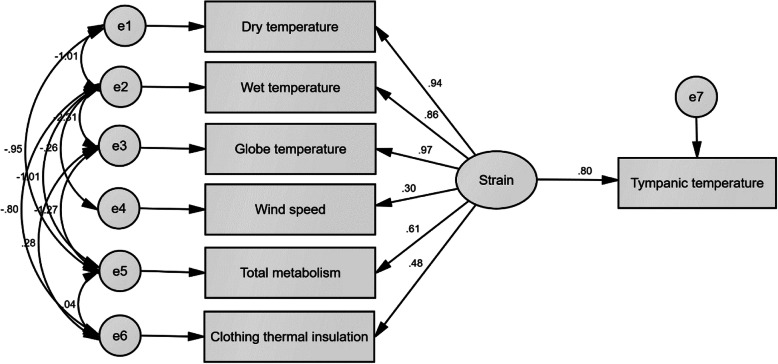
Theoretical model of the present study analyzed by SEM

Figure 1 shows the theoretical model of the present study analyzed using SEM. Based on this model, the heat strain produced by six main variables possesses factor loading of 0.80 on tympanic temperature. Moreover, Table 4 reports the direct and indirect effects of fundamental factors on the variations of tympanic temperature. The variables of the globe, dry, and wet temperatures with the coefficients of 0.77, 0.75, and 0.69 indicated the highest indirect effects on the tympanic temperature, respectively. Table 5 also represents the goodness-of-fit indices of the theoretical model of the present study. The results demonstrated that all obtained values of goodness-of-fit indices are in optimal ranges.

**Table 4:** Direct and indirect effects of fundamental factors on the variations of tympanic temperature

Variables	Direct effect	Indirect effect	*P* value
Dry temperature	0.939	0.752	*P* < 0.001
Wet temperature	0.857	0.686	*P* < 0.001
Globe temperature	0.966	0.774	*P* < 0.001
Air velocity	0.305	0.244	*P* < 0.001
Total metabolism	0.613	0.491	*P* < 0.001
Clothing thermal insulation	0.483	0.387	*P* < 0.001
Thermal strain	0.801	–	*P* < 0.001

**Table 5:** Goodness-of-fit indices of the theoretical model of the present study

Indices	Name	Fitness	Obtained value
Absolute fitness indices	Goodness-of-fit index (GFI)	> 0.9	0.991
	Adjusted goodness-of-fit index (AGFI)	> 0.9	0.947
Comparative fitness indices	Normed fit index (NFI)	> 0.9	0.992
	Comparative fit index (CFI)	> 0.9	0.998
	Incremental fit index (IFI)	0–1	0.998
Normed fit index	Root mean squared error of approximation (RMSEA)	< 0.1	0.043
	Normed Chi-square (X2/df)	1–3	1.364
*P* value		> 0.05	0.234

The environmental heat stress assessment risk (EHSRA) index was developed by indirect effect coefficients and normalized variables as follows:

3 $$ \mathrm{EHSRAI}=10\times \left[0.752\times \left(\frac{T_a-21.97}{26.23}\right)+0.686\times \left(\frac{T_w-12.10}{25.47}\right)+0.774\times \left(\frac{T_g-23.40}{39.30}\right)+0.244\times \left(\frac{V_a-0.0}{4.20}\right)+0.491\times \left(\frac{M_t-130}{360}\right)+0.387\times \left(\frac{I_c-0.50}{0.85}\right)\right] $$

Where *T*_*a*_ is dry temperature (degree of centigrade), *T*_*w*_ is wet temperature (degree of centigrade), *T*_*g*_ is globe temperature (degree of centigrade), *V*_*a*_ is air velocity (meter per second), *M*_*t*_ is total metabolism (Watts), and *I*_*c*_ is clothing thermal insulation (Clo). It is important to note that when dry temperature is lower than normal skin temperature (35 °C), the sign of air velocity coefficient changes from positive to negative because it decreases heat strain in these conditions.

**Fig. 2:**
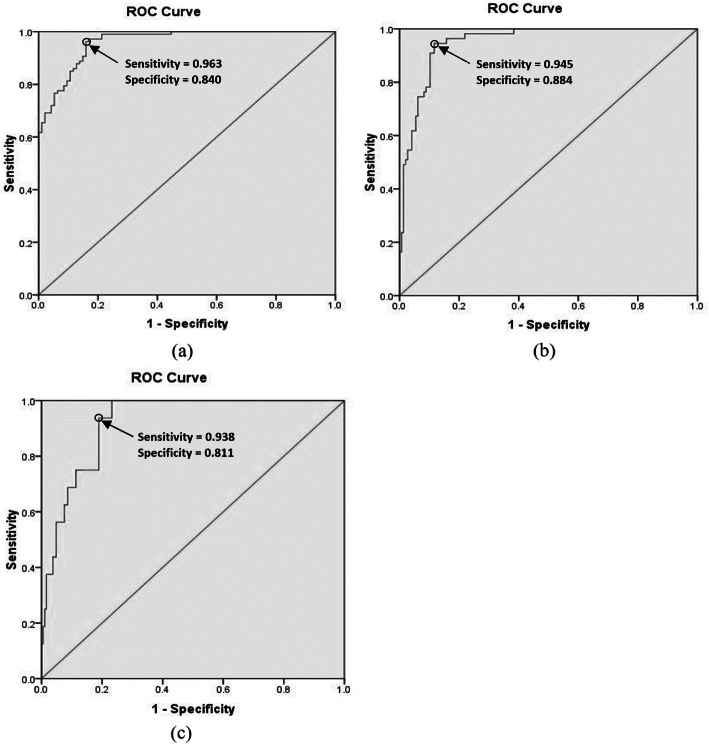
Receiver operating characteristic (ROC) curves related to **a** low and moderate risk zones, **b** moderate and high risk zones, and **c** high and very high risk zones

**Fig. 3:**
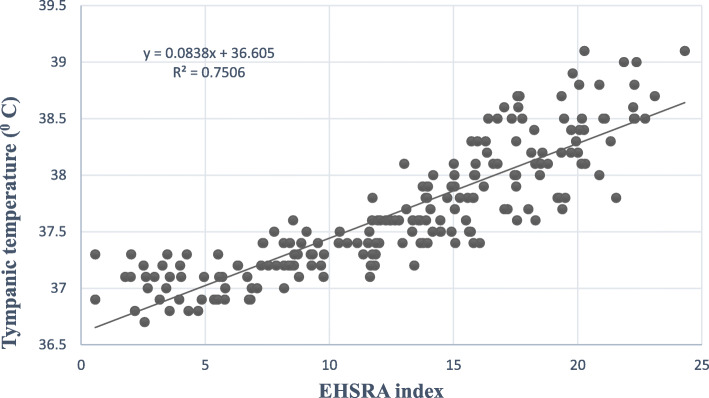
Linear regression curve between tympanic temperature and EHSRA index

Fig. 2a–c displays receiver operating characteristic (ROC) curves related to low and moderate risk zones, of moderate and high risk zones, and high and very high risk zones, respectively. Based on the results, optimal cut-off points of boundaries between low and moderate risk zones, between moderate and high risk zones, and between high and very high zones included 12.02 (sensitivity = 0.963 and specificity = 0.840), 15.88 (sensitivity = 0.945 and specificity = 0.884), and 17.56 (sensitivity = 0.938 and specificity = 0.811), respectively. Table 6 addresses the risk levels and equivalent scores of the EHSRA index. The area under of ROC curves (AUC), as a global summary statistic of diagnostic accuracy, in Fig. 2a, Fig. 2b, and Fig. 2c were 0.961 (95% CI 0.939, 0.983) (*P* < 0.001), 0.950 (95% CI 0.922, 0.978) (*P* < 0.001), and 0.921 (95% CI 0.875, 0.967) (*P* < 0.001), respectively. Furthermore, a linear regression analysis was performed between the developed index and tympanic temperature to investigate its validity. Figure 3 illustrates the linear regression curve between the tympanic temperature and the EHSRA index. The results showed that the EHSRA index justified 75% of the tympanic temperature variable (*R*^2^ = 0.75).

**Table 6:** The risk levels and equivalent scores of EHSRA index

Risk level	Equivalent score of EHSRA index
Low	Less than 12.02
Moderate	12.02 to 15.87
High	15.88 to 17.56
Very high	More than 17.56

## Discussion

To develop the EHSRA index, several important properties of a desirable indicator, including comprehensiveness, simplicity, and validity were considered. The index involved all fundamental effective variables in producing the heat strain, including dry temperature, wet temperature, globe temperature, air velocity, metabolism, and clothing thermal resistance. Results also indicated that heat strain produced by these fundamental parameters possess a direct effect with a factor loading of 0.80 on tympanic temperature. This reveals that the novel index can significantly predict the tympanic temperature. However, its prediction with higher accuracy requires more numbers of variables. Additionally, it was attempted to measure the extensive range of conditions in the industries and occupations. The values range of fundamental factors shows that the novel index can be applied in a variety of warm and hot workplaces.

Based on the results, the indirect effect coefficients of the globe temperature, dry temperature, wet temperature, air velocity, metabolism, and clothing thermal insulation variables were equal to 0.77, 0.75, 0.69, 0.24, 0.49, and 0.39, respectively. These coefficients were used to develop the EHSRA index. Dehghan et al also developed an observational-perceptual indicator using SEM, as named heat strain score index (HSSI). Coefficients of air temperature, wet temperature, air velocity, metabolism, and clothing thermal insulation consisted of 0.76, 0.65, 0.62, 0.64, and 0.31, respectively [20]. The coefficients of some parameters in the EHSRA index such as air temperature, wet temperature, and clothing thermal insulation obtained approximately similar to those in HSSI. However, variables of air velocity and metabolism showed significantly different coefficients. It may be due to observational-perceptual evaluation in the study of Dehghan et al. compared to environmental assessment in the present research. Also, a comparison of coefficients in the EHSRA index with those in some indices shows that there is a difference. In WBGT, as a valid heat stress indicator, wet temperature (0.7), global temperature (0.2), and dry temperature (0.1) possess the highest weights, respectively [21]. Liang et al. (2011) constructed a new environmental heat stress index using Cox regression. Weights of wet and dry temperatures calculated by 0.62 and 0.38, respectively [19]. Wet temperature compared to the globe and dry temperatures possess a lower variation range. For this reason, in WBGT and Liang et al indicator, the coefficient value of wet temperature is higher than that of the globe and dry temperatures. If the values of these temperatures are normalized and their variation range be equal, the coefficient of wet temperature decreases, and the coefficients of dry and globe temperatures increase. Also, high globe and air temperatures enhance thermal absorption through the convection and radiation mechanisms and produce a difficult condition to lose heat, while the high relative humidity diminishes the ability of sweat evaporation from the skin [22]. Hence, the moisture cannot affect thermal strain until the sweating mechanism is activated. For example, high humidity in low air and radiant temperatures cannot cause the heat strain. Perhaps for this reason, the coefficient of wet temperature obtained the value lower than that of dry and globe temperatures in the present study. The effect of parameters of air velocity, metabolism, and clothing thermal insulation in producing the heat strain also depends on stated environmental variables. Therefore, it seems that smaller impact coefficients of them are logical. Air velocity showed the lowest impact coefficient among fundamental factors. This parameter was often unstable in studied environments, which decreases its effects on the heat strain. Moreover, the impact of air velocity is controlled by the air temperature. So that if the dry temperature is lower than normal skin temperature, the air velocity decreases the heat strain, otherwise increases it [11].

After developing the EHSRA index, its calculated scores were categorized into four risk levels using ROC. The area under ROC (AUC) related to different risk levels consisted of 0.961, 0.950, and 0.921. The values of AUC greater than 0.90 indicate excellent diagnostic accuracy of curves [23]. Therefore, the scores can be easily and accurately interpreted based on this classification.

Finally, the validity of the EHSRA index, as the most important property of an indicator, was investigated. The results revealed that the presented theoretical model possessed good fitness and was acceptable. Furthermore, the results of linear regression analysis showed that the EHSRA index could justify 75% of the variations of the tympanic temperature. Several studies have investigated the validity of some known heat stress indices. Monazzam et al. concluded that there are *R*^2^ of 0.71 between WBGT and tympanic temperature under hot and humid conditions in the southern region of Iran. In this study, the predicted heat strain (PHS) index showed the highest correlation with body tympanic temperature (*R*^2^ = 0.76) [24]. Malchaire also studied the validity of PHS and results showed that *R*^2^ between this index and rectal temperature was equal to 0.66 [25]. In research of Falahati et al., *R*^2^ between WBGT and P4SR indices with human tympanic temperature were computed by 0.57 and 0.50, respectively [26]. However, these correlations are variable that may be due to the use of equipment with various accuracies, the measurement in diverse climatic conditions, and the difference in study design. The limitation of the present study involved a general evaluation of all environments by an index. For better accuracy, it is suggested that several indices developed to separately evaluate the environments with various climatic conditions. Moreover, because of ethical problems, it was not possible to use precise equipment such as rectal temperature for measuring core temperature in this study.

## Conclusion

In this study, a novel heat stress assessment index was developed using the main effective factors in producing the thermal strain. The results showed variables of globe temperature, dry temperature, and wet temperature possess the greatest effect coefficients, respectively. Based on the results, the novel index has appropriate validity. It is suggested that this index is applied and validated in various environments in future studies.

## Data Availability

All data generated and analyzed during this step of study are included in this published article.

## Abbreviations

WBGT: Wet bulb global temperature
PHS: Predicted heat strain
EHSRA: Environmental heat strain risk assessment
SEM: Structural equation modeling
ROC: Receiver operator curves
